# Development of a Three Dimensional Multiscale Computational Model of the Human Epidermis

**DOI:** 10.1371/journal.pone.0008511

**Published:** 2010-01-14

**Authors:** Salem Adra, Tao Sun, Sheila MacNeil, Mike Holcombe, Rod Smallwood

**Affiliations:** 1 Department of Computer Science, University of Sheffield, Sheffield, United Kingdom; 2 Centre for Cell Engineering, University of Glasgow, Glasgow, United Kingdom; 3 Department of Engineering Materials, University of Sheffield, Sheffield, United Kingdom; Cedars-Sinai Medical Center and University of California Los Angeles, United States of America

## Abstract

Transforming Growth Factor (TGF-β1) is a member of the TGF-beta superfamily ligand-receptor network. and plays a crucial role in tissue regeneration. The extensive *in vitro* and *in vivo* experimental literature describing its actions nevertheless describe an apparent paradox in that during re-epithelialisation it acts as proliferation inhibitor for keratinocytes. The majority of biological models focus on certain aspects of TGF-β1 behaviour and no one model provides a comprehensive story of this regulatory factor's action. Accordingly our aim was to develop a computational model to act as a complementary approach to improve our understanding of TGF-β1. In our previous study, an agent-based model of keratinocyte colony formation in 2D culture was developed. In this study this model was extensively developed into a three dimensional multiscale model of the human epidermis which is comprised of three interacting and integrated layers: (1) an agent-based model which captures the biological rules governing the cells in the human epidermis at the *cellular level* and includes the rules for injury induced emergent behaviours, (2) a COmplex PAthway SImulator (COPASI) model which simulates the expression and signalling of TGF-β1 at the *sub-cellular level* and (3) a mechanical layer embodied by a numerical physical solver responsible for resolving the forces exerted between cells at the *multi-cellular level*. The integrated model was initially validated by using it to grow a piece of virtual epidermis in 3D and comparing the *in virtuo* simulations of keratinocyte behaviour and of TGF-β1 signalling with the extensive research literature describing this key regulatory protein. This research reinforces the idea that computational modelling can be an effective additional tool to aid our understanding of complex systems. In the accompanying paper the model is used to explore hypotheses of the functions of TGF-β1 at the cellular and subcellular level on different keratinocyte populations during epidermal wound healing.

## Introduction

The immensity of the biological data provided by the sequencing of the human genome and the differing time scales (milliseconds to months) of the regulatory processes which take place within tissues give an indication of the complexities involved in understanding biological systems [**1**], [2****]. Computational modelling provides a powerful tool to handle such complexities and enhance our understanding of biological systems governed by sophisticated regulatory processes [**1**]–[3****]. It does this by offering a useful approach to processing and organizing a huge amount of complex biological data, allows the connecting of experimental results to fundamental biological principles and the opportunity to explore the roles of a single parameter in a complex biological system. The latter is particularly useful as it is often unattainable using *in vitro* or *in vivo* experimentation and it allows the identification of key parameters that play a central role in defining the overall behaviour of a biological system and it can lead to new and more informative experiments [**1**], [4], [**5**].

Among the various modelling approaches available, agent-based modeling (ABM) of biological systems is continuously gathering popularity among biologists. ABM is a flexible modeling approach which simulates the interactions of autonomous entities (agents) with each other and their local environment to predict higher level emergent patterns. ABM does not require any assumptions such as the smoothness or linearity of a system and the agents can represent any entities (e.g. atoms, molecules, cells or organs) regardless of their properties or domains. The simulation results of these models can be visualised and easily accessed by biologists, which promotes efficient cooperation between biologists and modellers [**5**].

The underpinning hypothesis behind the agent-based modelling of the cell is that the development of a complex tissue is crucially dependent on the coordination of relatively few cellular mechanisms [**1]**, [6], [7****]. Thus in our previous work in this area we used a rule set which describes several basic cellular behaviors of keratinocytes (proliferation, migration and differentiation) derived from the keratinocyte literature by abstracting the details of complex sub-cellular mechanisms to develop an agent-based colony formation model. This model was simulated using the agent-based modeling framework FLAME [**8**] then used to generate some predictions. These were tested in parallel using *in vitro* experiments and allowed us to explore hypotheses about how normal human keratinocytes (NHK) form colonies [**5**]. In this study, we have progressed this work in two directions. (1) We now model keratinocyte organization in 3D which allows us to simulate the formation of the human epidermis and (2) we have taken on a multi-scale modeling approach which allows us to link intracellular signalling rules (concerning one key growth factor, TGF-β1) and the emergent behaviours of NHK at the cellular level. The extended model comprises three interacting and integrated layers: (1) an agent-based model which captures the biological rules governing the cells in the human epidermis at the cellular level and includes the rules for the injury induced emergent behaviours, (2) a COPASI (COmplex PAthway SImulator) [**9**] model which simulates the expression and signalling of TGF-β1 at the sub-cellular level and (3) an improved and more realistic mechanical layer embodied by a numerical physical solver responsible for resolving the forces exerted between cells.

In the following sections we present (1) the integrated Agent-Based and COPASI Multiscale Model of the human epidermis, (2) an *in virtuo* experiment deploying the integrated model to grow a virtual piece of epidermis from a collection of stem cells, and (3) in Part B the derivation of the biological rules for TGF-β1 behaviour during epidermal wound healing are given in full and a series of experiments using the model to investigate the roles of this powerful cytokine during epidermal wound healing are presented and discussed.

## Materials and Methods

### The Agent-Based Modeling Framework: FLAME

FLAME (Flexible Large-scale Agent Modelling Environment) [**8**] (http://www.flame.ac.uk) is an agent-based modelling framework developed at the University of Sheffield and has previously been used to model the emergent behaviour of various biological systems (e.g. keratinocyte colony formation [**5**] and urothelial cells [**10**]). In this work, we use FLAME as the agent-based modeling framework to set up the 3D multiscale model of the human epidermis. One of the main features of FLAME is that it automatically generates parallel or serial executable code for the model specification defined by the user (in XML format). Moreover, in this work we will be deploying a special version of FLAME which includes an interface linking FLAME to COPASI (COmplex PAthway SImulator) [**9**]. COPASI is a software application used for simulating and analyzing biochemical networks, and it provides many useful functionalities such as stochastic and deterministic time course simulation, steady state analysis, metabolic control analysis and optimisation of arbitrary objective functions. For more information about COPASI, the interested reader is directed to http://www.copasi.org.

The interface linking FLAME and COPASI provides multiple capabilities which were used to set up the multiscale model of the human epidermis. These capabilities include: (1) Importing a SBML (Systems Biology Markup Langugage) model (through COPASI) to be used as part of the agent-based model in FLAME, (2) executing a COPASI model within FLAME to simulate time-course simulations (and solve ordinary differential equations (ODEs)) of biochemical networks taking place within the agents (e.g. intracellular signalling pathways) or the environment (e.g. intercellular reactions) and (3) updating a COPASI model based on the agents' memory values and vice-versa. Interfacing FLAME with COPASI provides an efficient multiparadigm modeling framework which links agent-based and mathematical (ODE) models. FLAME/COPASI can be loosely compared to CompuCell [11], another multiparadigm framework which links cellular automata and mathematical (ODE and PDE) models. CompuCell is widely used to model the interaction of the gene regulatory network with generic cell behaviours such as division or adhesion. More information about the different computational frameworks (including FLAME and CompuCell) available in the computational modeling community can be found on the Interagency Modeling and Analysis Group (IMAG) WIKI at: http://www.imagwiki.org/mediawiki/index.php?title=Computational_Frameworks_for_Modeling.

### The Multiscale Model of the Human Epidermis

**Figure 1:**
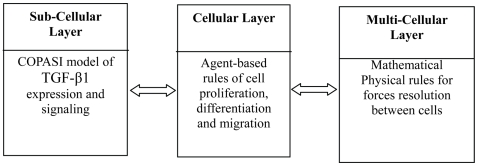
The three integrated layers of the multiscale model of the human epidermis.

The 3D computational model of the human epidermis consists of a further developed version of our previous NHK colony formation model [**5**] and is composed of three integrated and communicating layers (Figure 1).

The set of biological rules previously used in [**5**] is again deployed in the 3D multiscale model. These biological rules are mainly related to several basic cellular behaviours (i.e. proliferation, migration and differentiation) and all the subcellular details were abstracted away at this stage. However in the current study these basic cellular behaviours were controlled by variables instead of constants. The constants used in the original model were only used in this model as the default values for each of the model's parameters which in this model are now be regulated and controlled by mechanisms such as the TGF-β1 related internal decisions which include rules for the injury induced emergent behaviours. These TGF-β1 related decisions are governed by the “subcellular rules” defined in the COPASI model and will be described in the next sections.

The multiscale model comprises three types of agents: (1) Cell, (2) Tile and (3) Physical Solver Agents.

The cell agents (Table 1) represent normal human keratinocyte (NHK) cells and were each modelled as a non-deformable sphere (10 µm in diameter) governed by a rule set, and these cells were deemed capable of bonding, migrating, proliferating and differentiating. In the new model, the bonding, migration, differentiation and migration rules of the cell agents also embedded TGF-β1 functions which can stimulate different emergent cell behavior in specific scenarios such as the epidermal wound healing process.

**Table 1:** The Cell agent's functions and properties.

Cell Agent		
**Cell agent Sub-Types:**	Type 0 = Stem Cell	
	Type 1 = TA Cell	
	Type 2 = Committed Cell	
	Type 3 = Corneocyte	
**Virtual Shape**	Solid Sphere of diameter 10 µm	
**Biological rules (Cellular level)**	- Bonding Rules (E)	C Code implementations Simulated by FLAME
	- Cell Cycle and Proliferation Rules (E)	
	- Differentiation Rules (E)	
	- Migration Rules (E)	
	(E) = Embedded TGF-β1 Functions and injury induced emergent behaviours	
**Biochemical rules (Sub-cellular level)**	- TGF-β1 Expression	SBML model Simulated by COPASI
	- TGF-β1 Signalling	
**Physical Rules (Multi-Cellular level)**	- Resolution of the attractive and repulsive forces between cells	C Code Simulated by FLAME
	- Elimination of any existing cell overlap	
	The physical forces are resolved by calling the Physical solver agent to execute its function “resolve_physical_forces.c”	

The interaction of cells with the substrate was modeled by the use of tile agents as summarized in Table 2. The tile agents represented the surface of the extracellular matrix (ECM) on which the cells reside. In this research, the ECM surface was modelled as a user-defined flat modifiable surface composed of a certain number of tiles (20 µm×20 µm each) with a wall (100 µm high) around it. The tile agents were capable of changing their properties due to their interactions with cell agents. During epidermal wound healing for example, a NHK agent attached to or within certain proximity of a tile agent can behave differently depending on the type of its closest tile agent (proximity is measured as the euclidian distance between cell and tile agent centres). A provisional matrix tile agent promotes migration and inhibits proliferation of NHK. Secondary matrix enhances NHK proliferation and migration, while BM inhibits NHK proliferation and migration (simulated using rules at the cellular level). On the other hand, the NHK-provisional matrix interaction induces the remodeling of provisional matrix into secondary matrix, while the NHK-secondary matrix interaction induces the remodeling of secondary matrix into BM (simulated using rules at cellular level).

**Table 2:** The Tile agent's functions and properties.

Tile Agent		
**Tile agent Sub-Types:**	Type 4 = Basement Membrane	
	Type 5 = Secondary Matrix	
	Type 6 = Provisional Matrix	
**Virtual Shape**	Square surface (20×20 µm) on the the ECM	
**Biological rules**	ECM reconstruction (E)	C Code implementations Simulated by FLAME
	(E) = Embedded TGF-β1 Functions	

**Figure 2:**
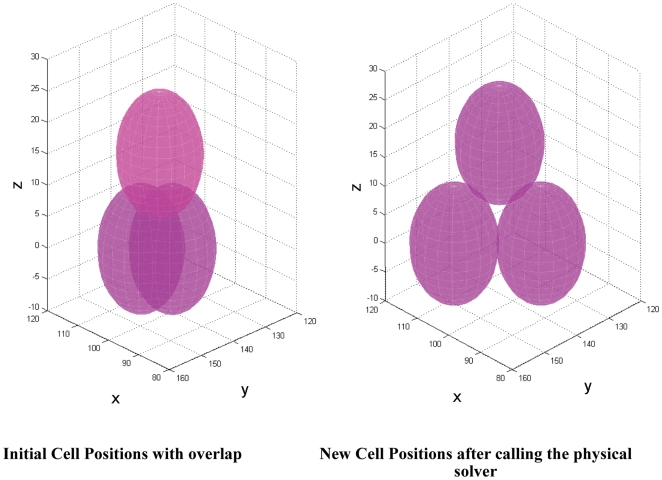
Physical solver agent applying repulsive forces to resolve the overlap between 3 cell agents.

Next we employed a single physical solver agent (Table 3) responsible for resolving forces between cells. This was also deployed in the 3D model. The physical solver consisted of a 3D version of the numerical solver used in [**10**] and was responsible for correcting any cell overlap resulting from mitosis or migration by applying repulsive forces which are proportional to the amount of overlap. The main reason behind the occurance of such physical cell overlap is that the biological and physical rules are executed serially instead of simultaneously as is the case in reality. In other words, at a certain model iteration (denoting 30 min in reality), cells first execute their biological rules (e.g. cell proliferation and migration) then the physical rules are executed. An example illustrating the role of the physical solver agent in resolving repulsive forces between three overlapping cells is presented in Figure 2. A detailed description of the deployed 3D physical solver can be found in Appendix S1. The relatively simple physical solver and the simple representation of the cell agents as non deformable spheres were mainly adopted to reduce the computational costs of running the multiscale model on a single desktop machine. Alternatively, and as future work, we might substitute these simple design choices with more realistic physical representations of the cell agents and with adaptive shapes for the cell dynamics by using the sub-element approach [12].

**Table 3:** The Physical Solver agent's functions and properties.

Physical Solver Agent		
**Cell agent Types:**	Type 7 = Physical Solver Agent	
**Virtual Shape**	Abstract (No shape)	
**Physical Rules**	- Resolve the attractive and repulsive forces between cell agents	C Code implementations Simulated by FLAME
	- Resolve any physical overlap between cell agents	

**Figure 3:**
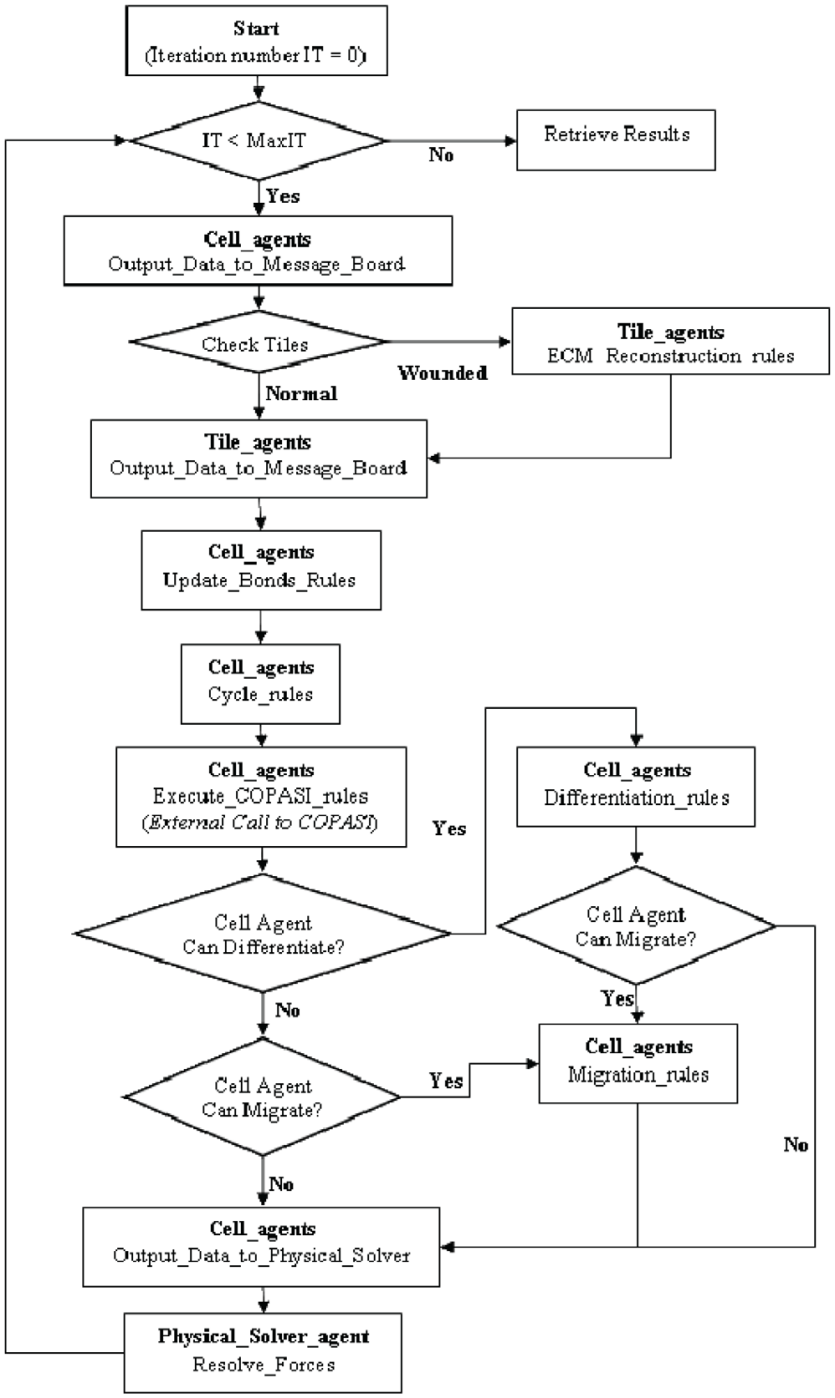
Flowchart of the multiscale model of the human epidermis. Pseudocode descriptions of the steps 1 to 12 are illustrated in Appendix S2. The physical solver (step 13) is described in detail in Appendix S1. The source codes for the entire model will be made available on http://www.flame.ac.uk.

A flowchart describing the multiscale model of the human epidermis is presented in Figure 3. At every iteration of the model, every cell and tile agent initially output their location, type (NHKs: stem cell, transit amplifying (TA) cell, committed cell or corneocyte; Tiles: provisional matrix, secondary matrix or basement membrane (BM)) and the concentration of TGF-β1 (either in its active form in the endosome of a cell agent or its latent form on the surface of a tile agent) to the message lists for other agents to read.

Each cell or tile agent then performs biological rules specific to its own position in the cell cycle or matrix remodeling process. Following this, agents decide whether to change to another agent type based on the differentiation rules or matrix remodeling rules. Cell agents also execute their migration rules. All rules are executed in the context of the agent's own internal state and its immediate environment as discovered through interrogation of the message lists. The time step for each iteration in the integrated model was set to 30 minutes.

In the next section, we present the TGF-β1 COPASI model and describe the modifications introduced to the biological rules previously used in our keratinocyte colony formation model [5]. Most importantly we will discuss how the expression and signaling of TGF-β1 at the intracellular level regulates the cellular behaviour of the cell agents and the ECM reconstruction rules of the tile agents.

### The TGF-β1 COPASI Model

TGF-β1 is a potent growth factor and has profound and paradoxical influences on epithelial cells in wound healing. The subcellular mechanisms of TGF-β1 are crucially important to understanding its functions in re-epithelialisation and effective research tools are needed due to the limitations of current biological models [**13]**–[16****]. In this research, extensive literature of TGF-β1 synthesis, expression, secretion, activation, signalling and function during re-epithelialisation were analysed carefully. The expression and signaling of TGF-β1 were simulated using COPASI, a process which was then integrated into the agent-based model of the human epidermis. Thus the behaviour of each cell agent was governed not only by the rule set at the cellular level, but also by the TGF-β1 subcellular mechanisms that were simulated with COPASI. Clearly TGF-β1 is not the only regulator of epithelial cell behaviour but the aim of this work was to create a flexible multiscale model that links intracellular signaling to a purely “agent-based” model of cell behaviour. The complexity of the suggested model can then be updated later on by including more regulators and intracellular signaling pathways. Other modeling paradigms -non agent-based - have been recently used to produce multiscale models that connect subcellular, cellular and macroscale elements and biological systems. The majority of such multiscale models are implemented using a mathematical or lattice based -cellular automaton- paradigm. Examples of such recent multiscale models include van Leeuwen *et al's* [17] integrative model of intestinal tissue renewal, Ramis-Conde *et al'*s [18] model investigating the effect of E-Cadherin-β-Catenin pathway in cancer cell invasion and Owen *et al'*s [19] multiscale model of vascular tissue growth.

The chemical reactions and coefficient factors used to simulate the expression and signaling of TGF-β1 in the COPASI model were largely based on a recently published study by Vilar et al [**20**]. In their research Vilar et al produced a SBML model of signal processing in the TGF-beta superfamily ligand-receptor network. Their model can be found online on the BioModels database of annotated published models (http://www.ebi.ac.uk/biomodels-main/BIOMD0000000101).

**Figure 4:**
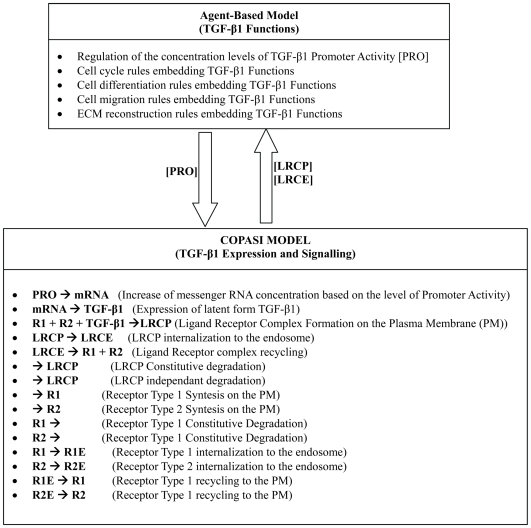
Communication channel between FLAME and COPASI. The source codes for the COPASI model described will be made available on http://www.flame.ac.uk.

In this study, we loaded Vilar et al's SBML model into COPASI, added some minor modifications and a few simple chemical reactions simulating the cascade of reactions leading to the production of the latent form of TGF-β1 based on information passed from the agent-based model, and created a two directional communication channel between COPASI and the agent-based model (Figure 4).

**Figure 5:**
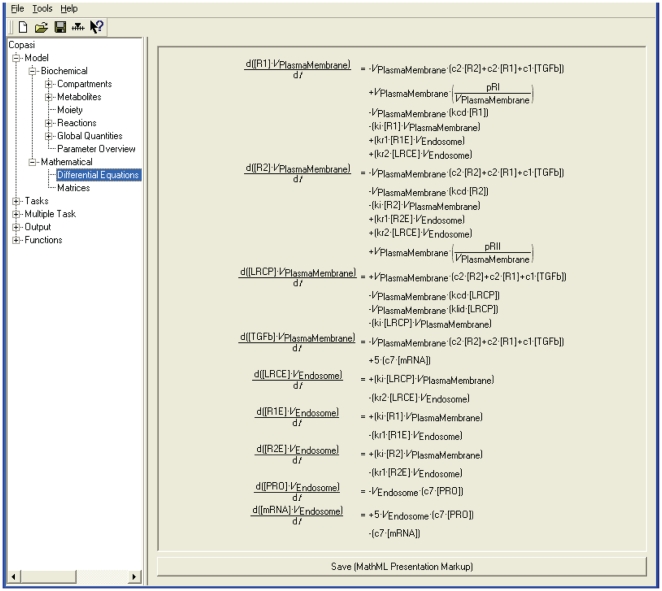
The list of ODEs used in the COPASI Model.

**Figure 6:**
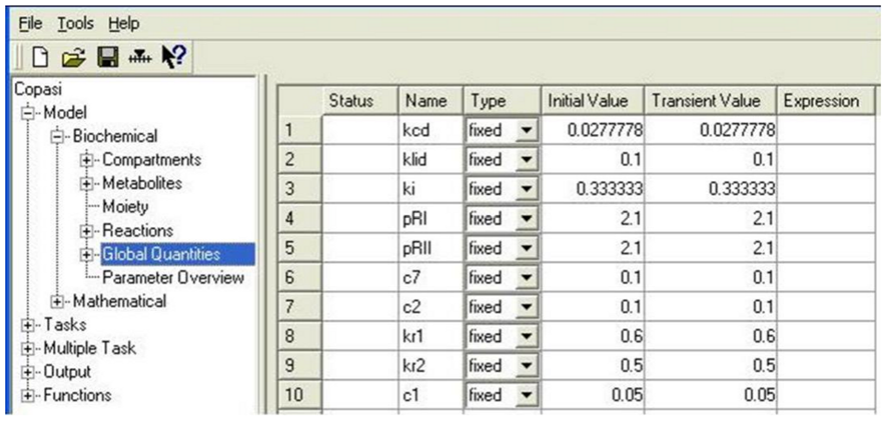
The list of global quantities used for solving the ODEs in the COPASI Model.

The agents' rule set which now embed TGF-β1 functions and the biochemical equations used in COPASI were based on the analysis of qualitative or semi-quantitative biological data about TGF-β1 synthesis, expression, activation, signaling and biological functions during re-epithelialisation. The suggested model however has been designed to be flexible and to be implemented in a way which allows these rules and equations to be further modified if required to reflect any new quantitative information on the biology that might reasonably be expected to emerge in the future. The initial concentrations used in the COPASI model for the different species involved in the TGF-β1 subcellular model and their initial emergent concentrations at the beginning of an epithelilal wound healing process are shown in Table 4. The set of ordinary differential equations and global quantities used in the COPASI model to simulate time course simulations of TGF-β1 synthesis and signaling are illustrated respectively in Figures 5 and 6. The assumptions used in the TGF-β1 simulations and in the cell level simulations and the relationships between the two are described in the next section.

**Table 4:** COPASI Model Parameters.

Time Unit (Min), Quantity Unit (mmol), Volume Unit (l),			
Task: Stochastic Time Course Simulation - Duration 30 Min–Number of Time Steps 100			
Species	Compartments	Initial Concentration in normal conditions (mmol/l)	Initial Concentration at the start of the wound healing process (mmol/l)
**R**eceptor type **1** on Plasma Membrane (**R1**)	**PlasmaMembrane**	**20**	**20**
**R**eceptor type **2** on Plasma Membrane (**R2**)	**PlasmaMembrane**	**20**	**20**
**L**igand **R**eceptor **C**omplex on **P**lasma membrane (**LRCP**)	**PlasmaMembrane**	**0**	**20**
**TGFβ1**	**PlasmaMembrane**	**10**	**100**
**L**igand **R**eceptor **C**omplex in **E**ndosome (**LRCE**)	**Endosome**	**0**	**20**
**R**eceptor type **1** in **E**ndosome (**R1E**)	**Endosome**	**10**	**10**
**R**eceptor type **2** in **E**ndosome (**R2E**)	**Endosome**	**10**	**10**
**mRNA**	**Endosome**	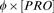	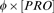

mRNA equation 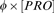 is defined in section “*Brief review of TGF-β1 and our simulation approach”*.

### Brief Review of TGF-β1 and Our Simulation Approach

In normal human epidermis, relatively low levels of TGF-β1 are expressed predominantly in the suprabasal, differentiating layers, suggesting it may have a role in maintaining the cessation of growth in the differentiating cells of epidermis [**21]**, [13****].

**Figure 7:**
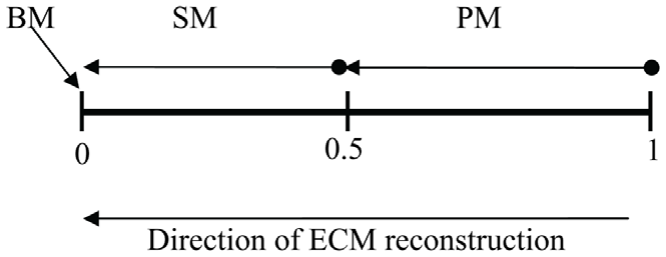
A Tile agent's position in the ECM reconstruction process. Each Tile agent has a memory variable “ECM_value” defined in the range [0, 1]. ECM_value = 0→Tile agent is a BM tile, 0.1≤ECM_value≤0.49→Tile agent is SM tile, and 0.5≤ECM_value≤1→Tile agent is PM tile.

During re-epithelialisation, the expression of TGF-β1 is induced by various ECM components. (See Part B for the detailed justification of these biology rules derived from an extensive literature on TGF-β1). For example, the disruption of BM can dramatically enhance TGF-β1 promoter activity, TGF-β1 mRNA level and thus the expression of latent TGF-β1 in keratinocytes, which can be futher up-regulated by active TGF-β1 itself [**13**]. Meanwhile, TGF-β1 induces the secretion of various ECM proteins in an autocrine manner, contributing to the establishment of more physiological cell-ECM interactions, which subsequently downregulates the expression of TGF-β1. There is a feedback loop mechanism to keep the balance between ECM remodelling and TGF-β1 synthesis [**13]**, [22]–[25****] and the expression of TGF-β1 is thus confined within a certain area in the wound bed [**13]**, [22], [25****]. In the integrated model, stem and or TA cells express TGF-β1 when (1) they are stratified and a certain distance away from the matrix surface, (2) in contact with provisional matrix, (3) under the regulation of TGF-β1, which can be down regulated by the presence of secondary matrix and BM components. All these TGF-β1 expression regulation signals are detected by cell agents through interrogation of the message lists and passed to the TGF-β1 COPASI model. The subsequently induced subcellular mechanisms from the activation of TGF-β1 promotor acticity, induction of mRNA to the synthesis of the latent form of TGF-β1 were simulated by COPASI. The promoter activity level (PRO) increases depending on three essential factors: (1) The presence of provisional matrix tiles (PM) or secondary matrix tiles (SM) which exist in wounded area, (2) the concentration of active TGF-β1 ligand receptor complex (LRCP) on the cell membrane and (3) the cell strafication distance (D) as shown in the following equations:

(1) 
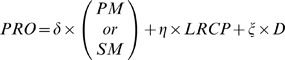
 OR (2) 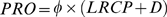 if a cell is on a BM; Where δ ( = 10), φ ( = 0.4), η( = 1) and ξ( = 1) are qualitative coefficient factors related to the above TGF-β1 expression signals and BM, SM and PM are real values in the range [0, 1] denoting a tile's agent position in the ECM reconstruction process (Figure 7).

The mRNA level (mRNA) depends on the concentration of promoter activity based on the equation: mRNA = Φ * PRO, Where Φ ( = 5) is a transcription coefficient factor and defined based on current qualitative research. The expression of TGF-β1 latent complex in its turn depends on the level of mRNA as shown in the following equation: TGF-β1 = Ω * mRNA; Where Ω ( = 5) is a translational coefficient factor and defined based on current qualitative research. The expression of TGF-β1 was simulated for 30 mins in each iteration and the synthesised TGF-β1 was directly deposited onto the matrix undermeath the cells or onto the membrane on neighbouring cells. The highest production of TGF-β1 from each cell was set as 200 millimolar/30 mins. The life cycles of active and latent TGF-β1 were modifiable parameters and both were set as 48 hours, that is, both types of TGF-β1 could degrade within 48 hours based on *in vitro* research [**21**].

In order to reach the appropriate target cell in biologically relevant concentrations at the correct time, various methods have been employed to oppose entropy, augment the process of diffusion and concentrate and store TGF-β1 in the ECM [**25]**–[34****]. Firstly, TGF-β1 is synthesized as biologically inactive large latent complexes composed of a latent TGF-β binding protein (LTBP) covalently bound to the latency associated protein (LAP) and TGF-β. Due to the covalent association between LTBP and specific ECM components such as fibrinectin (Fn), most of the secreted latent TGF-β1 is concentrated and fixed in the ECM [**13]**, [27]–[32****]. The subsequent retrieval of latent TGF-β from ECM and its activation is a critical regulatory step in the action of TGF-β1 [**13]**, [26], [28], [33], [35], [36****]. There are two main biological mechanisms to release active TGF-β1: (1) a conformational change of latent TGF-β1 [**35]**, [37****] by its direct interaction with cell surface receptors or proteins such as integrins (e.g. αvβ1, αvβ8, αvβ6) and thrombospondin (TSP)-1; (2) proteolysis of LAP by proteases such as plasmin and MMP [**32]**, [35], [38****]. During wound healing, the αvβ6 induced TGF-β1 liberation and activation from ECM plays an important role [**35]**, [37], [39****], which requires close associations between αvβ6, LAP, LTBP and Fn [**13]**, [25], [27]–[32], [35], [39****]. Both TGF-β1 and Fn are parts of a feed forward loop regulating ECM formation and TGF-β1 activation, since Fn plays important roles in TGF-β1 storage and activation, meanwhile TGF-β1 induces the synthesis and incorporation of Fn into the ECM. This feed forward mechanism is organized mainly by activating cells at the time of TGF- β activation within a confined area [**32]**, [39****] as both TGF-β expression and avtication can also be suppressed by a remodelled ECM [**25]**, [40****]. Consequently, the depositation and activation of TGF-β1 are also spatially restricted within certain area by several mechanisms [**13]**, [22], [33****]. A notable exception to the activation process is that TGF-β1 is abundantly released by platelets and macrophages at the injury site in active form [**22]**, [26], [28], [33], [41****].

As the literature analysis suggested, a random diffusion equation could not simulate the process of TGF-β1 concentration and fixation into ECM. Thus the amount of latent TGF-β1 (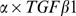) deposited into the ECM was simulated by defining α at various level to reflect the specific concentration and fixation mechanisms. In provisional matrix the value of α ( = 90% of produced TGF-β1) is very high due to the presence of ECM proteins such as Fn, but it is very low in secondary matrix and BM (1%) due to the remodelling of ECM components. The TGF-β1 in the matrix was only activated by the cells that directly contacted with the ECM matrix and the activated TGF-β1 (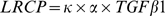) on the cell membrane was simulated by defining κ as 90% to reflect the αvβ6 induced specific activation mechanism on the cell membrane.

TGF-β1 regulates cellular processes mainly by binding to three high-affinity cell-surface receptors known as types I, II, and III (TbRI, II, III). TbRIII functions by binding and transferring TGF-β1 to TbRII, but TGF-β1 can bind to TbRIIs with or without the help of TbRIIIs. Once activated by TGF-β1, TbRIIs recruit, bind and transphosphorylate TbRIs. The TbRI then activates the downstream effectors (i.e. Smad2 and Smad3) by phosphorylation. The activated Smad proteins form complexes with the common Smad mediator, Smad4, and then translocate to the nucleus, where the Smad complexes regulate transcription of TGF-β1 target genes in conjunction with various transcriptional or co-transcriptional regulators [**25]**, [26], [42], [43****]. Other non-Smad signaling pathways can also be activated by TGF-β in a context-dependent manner [**42]**, [43****].

A peculiarity of the TGF-β pathway is that receptors are constitutively internalized, even in the absence of ligand. Differential kinetics in biosynthesis, degradation and trafficking of TbRI and TbRII can modulate TGF-β signaling [**44]**, [25****], which has already been simulated using COPASI and published [**20**]. In this study, the published TGF-β signaling COPASI model was directly adapted as part of our own COPASI model. In the integrated model, the activated TGF-β1 (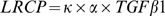) on the cell membrane can be used by the COPASI model of TGF-β signaling to simulate the level of ligand-receptor in the endosome (LRCE) as described elsewhere [**20**], which is then used by the TGF-β1 functions embedded in the cell cycle, migration, differentiation and bonding rules discussed in the following section.

TGF-β1 is a pleiotropic growth factor with both growth-promoting and growth-suppressive activities depending on circumstances, including concentration, target cell type and context [**13]**–[15], [26], [34], [42], [45], [46****]. As previously stated the role of TGF-β1 in the re-epithelialization process appears paradoxical and is investigated in the second part of this work (B). It is a strong inhibitor of keratinocyte proliferation [**34]**, [46****], which interferes with the supply of extra keratinocytes to cover the wound bed. On the other hand, it promotes cell migration by inducing the expression of integrins and proteases etc [**47]**–[49], [15], [43], [22], [13****]. TGF-β1 also regulates the behaviour of keratinocytes by inducing the expression of various ECM proteins such as tenascin, thrombospondin, fibronectin, vitronectin, collagen and several proteoglycans etc. [**22]**, [13], [25], [16], [26], [42], [32], [48****]. The controversial functions of TGF-β1 on wound closure have been confirmed by various biological models [**15]**, [50], [51], [33], [43], [22****]. In this integrated model, the influence of TGF-β1 on the expression of integrins, proteases and the proliferation of keratinocytes depend on the level of ligand-receptor complex in the endosome (LRCE).

### Development of the Agent and COPASI Based Integrated Model

First of all, biological rules of the emergent behaviours of NHK induced by various injury signals were derived and incorporated into the keratinocyte colony formation model to establish the 3D model of the epidermis. The TGF-β1 expression and signaling were then simulated using COPASI and integrated with the model. Most of the biological rules used in the previous keratinocyte colony formation model [**5**] were slightly altered in this work to capture cell behaviour in the human epidermis as opposed to 2D culture.

Briefly, NHK stem cells can attach to the surface of ECM, proliferate, form tight colonies, and automatically control the size of the stem cell colony. When the stem cell colony reaches a certain size, the stem cells on the colony edge will differentiate into TA cells. TA cells can migrate, divide, stratify and control the size of the TA cell colony due to the auto-regulation mechanisms (as described in [**5**]). When TA cells are a certain distance away from stem cells, they will differentiate to committed cells. Committed cells gradually lose their nuclei and further differentiate into corneocytes [**5**]. The new biological rules, the modification of the original rules and the simulation of TGF-β1 in the integrated model are described in the following sections.

#### 1. Cell migration embedding TGF-β1

Within hours after the wounding of adult skin, keratinocytes at the wound margin start to flatten, elongate, develop pseudopod like projections of lamellipodia and migrate toward the denuded area [**52]**–[54****]. The essential mechanisms responsible for the motility or flux of cells including epithelial cells are mitotic activity, cell active movement, cell-cell and cell-substrate interactions [**55]**, [56****]. In the 3D model of the epidermis (which will also be referred to as the re-epithelialisation model in the second part of this paper), there are attractive and repelling forces between different agents as in the previous model. The attractive forces simulate cell-cell and cell-substrate bonds, and are applied when the respective bodies (cell-cell or cell-substrate) and are within 5 µm of one another, which keeps keratinocytes in the coherent cell sheet and the epidermis to the surface of the ECM. In the integrated model, however, the cell-cell and cell-substrate bonds were simulated using variables instead of constants, which can be regulated by the function pathway of the TGF-β1 COPASI model. The ligand-receptor level in the endosome (LRCE) simulated by the TGF-β1 signalling pathway in COPASI was used by the TGF-β1 function pathway to further simulate the expression of integrin (IN) and proteases (PR) according to the equation: 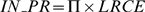, where Π is a coefficient factor defined as 0.5.

Based on the expression of integrin (IN) and proteases (PR), the cell-cell bonds (CCB) and cell-substrate bonds (CSB) will be modified by the physical model according to the following equations: where CCB0 and CSB0 are the default values (i.e. the original cell-cell bond and cell-substrate bond used in the previous keratinocyte colony formation model).



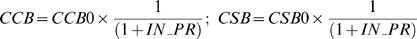



Epidermal cells generally maintain cell-cell contacts and migrate as a coherent sheet, rather than as single free entities [**57]**–[61], [22****]. Moreover, the keratinocytes in normal wound healing process are regulated to migrate toward the denuded area on the provisional matrix due to various mechanisms such as cytokines and ECM proteins. For example, keratinocytes in the migrating front deposit laminin 5, which serves as a track to allow subsequent keratinocytes to migrate [**52**]. Thus two alterations were made to the active migration of TA cells used in the previous model. Firstly, TA cells can migrate actively at the same rate (1 µm/minute) as in the previous model provided they keep contacts with other cell agents. Secondly, a high tendency for the TA cells to migrate toward or on the provisional matrix is defined for active migration of TA cells.

#### 2. Cell proliferation embedding TGF-β1

Although epidermal cells are able to generate local niches and control the cell number due to various autoregulation mechanisms [**62]**, [63], [3****], environmental factors such as injury signals play an important role in regulating the proliferation of basal cells. From about 12 hours to 1–2 days after wounding and some hours after the onset of migration, there is a marked increase in mitotic activity in the basal cells a small distance away from the wound edges [**49]**, [53], [64****], providing an extra source of basal cells to supplement of the advancing and migrating epithelial tongue [**52**]. As well as the auto-regulation mechanism, the influence of the ECM matrix and of TGF-β1 on cell proliferation were simulated expilicitly, while the influence of other factors on cell proliferation were modelled implicitly in the re-epithelialisation model. For example, to simulate the bombardment of various injury related signals on cell proliferation, the sizes of the auoregulated stem and TA colonies were simply simulated to be bigger than that in the previous model. The cell proliferation rates of both stem and TA cells were also simulated using different division probabilities instead of a single probability (Table 5). Moreover, depending on the ligand-receptor level in the endosome (LRCE) simulated by the TGF-β1 signalling pathway the cell proliferation rate (PRR) of stem and TA cells was regulated.

**Table 5:** The different division probabilities (DP) adopted in different scenarios.

DP	Scenario
90%	Stem or TA cell on top of a provisional matrix tile or in wound bed
40%	TA and Stem Cells in normal epidermis (no contact inhibition)
5%	Stratified TA cells in the wound bed
2%	Contact inhibited TA cell in normal epidermis
1%	Contact inhibited Stem cell in normal epidermis

#### 3. Cell differentiation embedding TGF-β1

In the integrated model, the differentiation signals such as ceramide and FAS-L were only kept between committed cells and corneocytes to simulate the homeostasis of human epidermis, while the other differentiation rules in the previous model were kept unaltered. The code for this model will be made available from: http://www.flame.ac.uk.

## Results

### A Virtual Epidermis Created Using the Integrated Model

**Figure 8:**
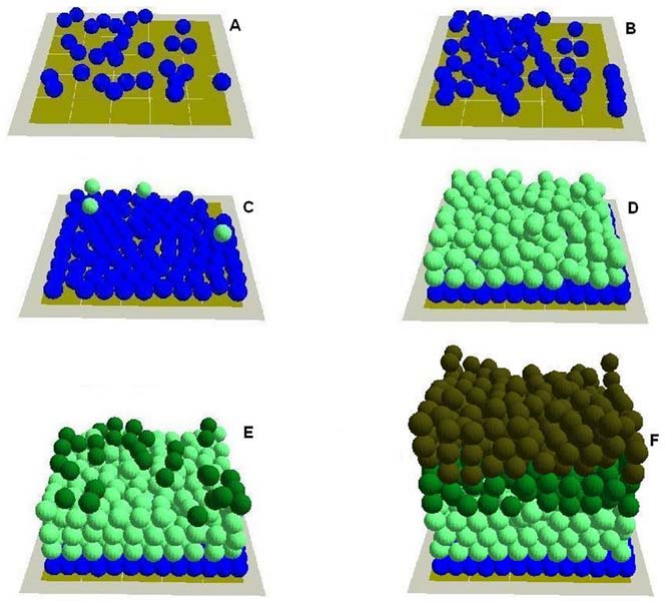
Creation of a virtual epidermis using the agent and COPASI based multi-scale model. In the integrated model (A–F) different colours were used to represent the different keratinocyte cells: keratinocyte stem cells (blue), TA cells (light green), committed cells (dark green), corneocytes (brown), BM tile agent (light brown).

The agent and COPASI based integrated model was used to create a virtual epidermis for model validation. In part B of this paper, the same model is used for *in virtuo* exploration of several hypotheses related to epidermal wound healing process. A defined number (30) of stem cells were randomly seeded on the virtual BM surface (100 µm×µm). The stem cells proliferated and formed a stem cell colony on the BM (Figure 8a, b), which gradually expanded and occupied the whole BM surface. When the stem cells started to become confluent on the BM surface, some of the stem cells started to stratify and differentiate into TA cells (Figure 8c, d). TA cells later on migrated, proliferated, stratified and differentiated into committed cells (Figure 8e).

Committed cells withdrew from the cell cycle and further differentiated into corneocytes. Finally, a virtual epidermis with stratified squamous epithelium composed mainly of keratinocytes at different stages of proliferation/differentiation was created by the integrated model (Figure 8f). The previously described process of growing a virtual piece of epidermis using the integrated model is also illustrated in the attached Movie S1. The simulation which led to the production of the virtual epidermis illustrated in Figure 8f was executed on a single desktop machime running Windows XP on an Intel Pentium 4 (2.26 GHZ) single core processor and 1 GB of RAM memory. The simulation was executed several times for testing purposes, each time ending with roughly 1500 agents and lasting between 15 to 20 hours. Further testing of the intact virtual epidermis indicated that the cell proliferation was mainly limited to the stem and TA cell area that is close to the BM at relatively very low level. The 3D structure of the stratified squamous epithelium was also maintained and was not influenced by further simulation runs indicating the validaty of the model in terms of its capability to simulate the basic properties of an intact epidermis. Accordingly this virtual epidermis model was deemed suitable for the *in virtuo* investigation of epidermal wound healing.

### Proposed Experiments Investigating the Roles of TGF-β1 during Epidermal Wound Healing

Following the development and setting-up of the model described in this paper, in Part B we investigate the profound roles of TGF-β1 during epidermal wound healing and explore different hypotheses about the apparently paradoxical roles of this growth factor. Specifically we explore

- Typeface="12";the regulation of epithelial formation using the integrated model
- Typeface="12";TGF-β1 expression in the different parts of an intact epidermis
- Typeface="12";*In virtuo* investigation of epidermal wound healing with different wound sizes
- Typeface="12";cell proliferation and migration during epidermal wound healing
- Typeface="12";*In virtuo* investigation of TGF-β1 signaling during epidermal wound healing

## Discussion

Complex biological regulatory processes on the sub-cellular scale impact on cell behaviour at the cellular level and then at the multicellular level. However there are relatively few cell ‘behaviours’ - cell adhesion, migration, proliferation and differentiation - that at a gross level influence the formation of complex tissues [**65]**, [66], [6], [2], [7****]. An agent-based modeling approach can be used to reduce the complexity of a biological system by abstracting away the micro-level or subcellular details to produce a global view of the investigated biological system, which then allows the testing of hypotheses and the designing of new informative experiments. Using this computational approach we previously developed an agent based keratinocyte colony formation model, which improved our understanding of the interactions between various keratinocytes and how they self-regulated. This then led to testing of hypotheses with the design of an *in vitro* experiment to investigate the influence of cell proliferation and migration on scratch wound healing [**5**].

However as we had deliberately abstracted all subcellular details, any *in virtuo* investigations of the regulation of a specific subcellular pathway on multicellular behaviours or tissue morphogenesis was clearly not possible in this agent based model. The regulation of epidermal homeostasis involves a complex interplay between different exogenous and genetic mechanisms. Cells actively change their properties and behaviour as a consequence of internal decisions or “subcellular rules” that are encoded in the genetic information which allows them to respond “appropriately” to external signals such as soluble factors or insoluble ECM. Therefore, it will be very useful to investigate specific subcellular mechanisms if the model can combine the description of a cell with a description of the “subcellular rules” that dictate the change of its behaviour or parameters [**2]**, [4], [67****]. While there are many such regulatory factors we have selected TGF-β1 to explore this approach as it is recognized as being a key regulatory factor of tissue formation.

Our aim in this study was to develop an integrated model that would allow us to link specific subcellular mechanisms (in this case related to TGF-β1) into the cellular level rules of the agent based model to investigate the influence of TGF-β1 on epidermal wound healing.

This study describes the development of a multiscale integrated model of a human epidermis. An agent-based keratinocyte colony formation model previously established in our group [**5**] was extended to a multiscale 3D model of a virtual epidermis. This new model includes new biological rules about the emergent responses of keratinocytes to various wound injury signals abstracted from the extensive published literature. At this stage the rules used were still at the cellular level governing the basic cellular behaviours of cell proliferation, migration and differentiation. The subcellular details of TGF-β1 expression, signaling and regulation were then simulated using COPASI and integrated into this model.

This integrated model will allow us to simulate various cell-cell and cell-ECM interactions at the cellular level, and to explicitly investigate the subcellular regulation of TGF-β1 on keratinocyte behaviour. In this paper, the model is introduced and described in detail. The model's validity was also checked by deploying it to create a virtual epidermis from scratch. The validity of the basic biological rules at the cellular level and the subcellular mechanisms used in the COPASI were tested by qualitatively comparing the simulation results of an intact epidermis with published research. The results demonstrated that the model successfully simulated many of the described behaviours of keratinocytes and TGF-β1 subcellular mechanisms, suggesting the validity of the model.

In summary this integrated model provides a flexible framework which can be used by biologists to investigate the effect of different key parameters and hypotheses of epidermal tissue formation. It can be deployed in future studies to investigate the roles and functions of other growth factors (such as epidermal growth factor EGF) and signaling pathways (such as the “wnt” signaling pathway) with the aim of producing a more realistic virtual cell. The model's flexibility also allows for future easy modification of the biological parameters to reflect new biological research. The three dimensional aspect of the integrated model also makes it easier for biologists to visualize the results and make qualitative and quantitative comparisons with biological data collected from *in vitro* and *in vivo* experiments. The linkage between FLAME and COPASI deployed in the integrated model also provides an innovative framework which facilitates the incorporation of published and curated intracellular signaling pathways into an agent based model. The proposed model can be simulated on any computer achitecture and can be scaled using FLAME to run on multiprocessors and high performance computers.

Applications of this model to explore epidermal wounding and repair are given in the accompanying paper B.

## Supporting Information

Appendix S1 The Physical Solver
(0.13 MB DOC)

Appendix S2 Model Pseudocodes
(0.05 MB DOC)

Movie S1 Growing, wounding, then healing a piece of virtual epidermis. In this movie, we illustrate how the integrated model is used to grow a piece of skin tissue from a collection of stem cells. Once the virtual epidermis is created, a scratch is introduced to simulate a small wound taking place. After few more iterations of the computational model, the wound is completely healed.
(5.95 MB WMV)
