# Supplementary material for: Development of a Three Dimensional Multiscale Computational Model of the Human Epidermis
Source: PLoS One. 2010 Jan 14;5(1):e8511. doi: 10.1371/journal.pone.0008511 (PMC2799518; doi:10.1371/journal.pone.0008511)
Supplement: Appendix S1 — The Physical Solver (0.13 MB DOC) [file pone.0008511.s001.doc]

**Appendix S1: The Physical Solver**

At the current stage of development, the physical realisation of the cells and their interactions is very simplistic. The cells are rigid spheres. They are not confined to a plane, i.e. multilayer of cells is modelled. Cell movement and mitosis events during each agent-based iteration will result in some degree of overlap between neighbouring cells. In addition, the edges of neighbouring cells that are bonded to one another may not be contiguous.

The acceleration induced in the ith cell, by the force exerted by its n neighbours is given by Newton’s second law, with damping:

(1.1)

where $m_{i}$ is the mass of the cell, $c_{i}$is its damping constant and $u$ is its displacement. The mass of the cell is calculated from the length of its radius *r*, which is information retained in the computational data structure representing each cell. As the cells interact in three dimensions, this is based on the volume of the cell, assuming a unit density:

$m_{i}=\frac{4}{3}\pi r_{i}^{3}$ (1.2)

The damping constant, $c_{i}$ is proportional to the mass of the cell:

$c_{i}=\alpha m_{i}$ (1.3)

where the proportionality constant $\alpha$is assigned according to the global location of the cell. By default, α=0.1, but cells close to the edge of the model are given higher damping factors to inhibit their movement and prevent them being pushed off the edge of the model. Damping factors in the *x*, *y* and *z* direction can be manipulated independently, thus a cell may be more easily pushed in one direction than the other.

All agent locations and radii are passed from the agent model to the physical model. For each ‘physical’ iteration, a check is made to identify with which nearby cells an agent is overlapping. In addition, a list of the flags of other agents to which it has formed an intercellular bond is passed, and an attractive force is assumed to exist between the latter in order to counteract any tendency for bonded cells to be forced apart. Forces are assumed to be zero between agents that are neither overlapping, nor bonded.

The repulsive force exerted on the cell *i* by neighbour *j* is defined to be proportional to the overlap or edge separation $O_{ij}$ between the cells.

$F_{ij}=k_{ij}O_{ij}$ (1.4)

where $k_{ij}$ is an arbitrary ‘stiffness’ constant defined to be equal to the inverse of the separation of the centres of cells *i* and *j*. If cells *i* and *j* have radii $r_{i}$ and $r_{j}$ and are located at $(x_{i},y_{i,}z_{i})$ and $(x_{j},y_{j,}z_{j})$ respectively, then:

$O_{ij}=\sqrt{\left(x_{j}-x_{i}\right)^{2}+\left(y_{j}-y_{i}\right)^{2}+\left(z_{j}-z_{i}\right)^{2}}-(r_{i}+r_{j})$ (1.5)

Note that the signs of the constants are constructed in such a way that cells experience a repulsive force from overlapping neighbours, and an attractive force from bonded neighbours that may be separated by a small distance.

Assuming the vector $i\to j$ is orientated at angle $\theta$ with respect to the *x* axis and at angle$\phi$ with respect to the *z* axis, resolving into three orthogonal directions and substituting into (1.4) gives:

$F_{ij(x)}=k_{ij}\left\{\left(x_{j}-x_{i}\right)-(r_{i}+r_{j})\cos\theta\sin\phi\right\}$ (1.6a)

$F_{ij(y)}=k_{ij}\left\{\left(y_{j}-y_{i}\right)-(r_{i}+r_{j})\sin\theta\sin\phi\right\}$ (1.6b)

$F_{ij(z)}=k_{ij}\left\{\left(z_{j}-z_{i}\right)-r_{i}\cos\phi-r_{j}\cos\phi\right\}$ (1.6c)

The co-ordinates of cell *i* at time *t* are the co-ordinates $(x_{Oi},y_{Oi},z_{Oi})$at the previous time step plus the displacement $u_{xi,}u_{yi,}u_{zi}$during the current time step, so equation 1.6a can be written:

$F_{ij\left(x\right)}=k_{1ij}\left\{\left(x_{Oi}-x_{Oj}\right)+\left(u_{xi}-u_{xj}\right)\right\}-k_{2xij}\left(r_{i}+r_{j}\right)$ (1.7)

where $k_{1}$ and $k_{2x}$ are constants depending on the location of the two cells:

$k_{1_{ij}}=\frac{1}{\sqrt{\left(x_{j}-x_{i}\right)^{2}+\left(y_{j}-y_{i}\right)^{2}+\left(z_{j}-z_{i}\right)^{2}}}$ (1.8a)

$k_{2x_{ij}}=\frac{\cos\theta\sin\phi}{\sqrt{\left(x_{j}-x_{i}\right)^{2}+\left(y_{j}-y_{i}\right)^{2}+\left(z_{j}-z_{i}\right)^{2}}}$ (1.8b)

Equations 1.6b and 1.6c can be similarly rewritten, but in this case:

$k_{2y_{ij}}=\frac{\sin\theta\sin\phi}{\sqrt{\left(x_{j}-x_{i}\right)^{2}+\left(y_{j}-y_{i}\right)^{2}+\left(z_{j}-z_{i}\right)^{2}}}$ (1.8c)

and

$k_{2z_{ij}}=\frac{\cos\phi}{\sqrt{\left(x_{j}-x_{i}\right)^{2}+\left(y_{j}-y_{i}\right)^{2}+\left(z_{j}-z_{i}\right)^{2}}}$ (1.8d)

The value of the differential terms in equation (1.1) at time *t*, can be written as difference equations:

(1.9a)

(1.9b)

where $u_{i,t-\partial t}$ and $u_{i,t-2\partial t}$ denote the displacement of the *ith* cell evaluated at the two previous time points. Substituting equations 1.7 and 1.9 a and b into 1.1 gives the complete equation of motion for cell *i* in the x direction:

$m_{i}\left\{\mathop{\frac{u_{xi,t}-2u_{xi,t-\partial t}+u_{xi,t-2\partial t}}{\partial t^{2}}}\limits^{..} \right\}+\alpha_{i}m_{i}\left\{\frac{u_{xi,t}-u_{xi,t-\partial t}}{\partial t}\right\}=\sum_{j=1}^{n} \left[k_{1ij}\left\{\left(x_{Oi}-x_{Oj}\right)+\left(u_{xi}-u_{xj}\right)\right\}-k_{2xij}\left(r_{i}+r_{j}\right)\right]$ (1.10a)

Similar equations can be assembled to describe the y and z displacements of every particle in the model.

$m_{i}\left\{\mathop{\frac{u_{yi,t}-2u_{yi,t-\partial t}+u_{yi,t-2\partial t}}{\partial t^{2}}}\limits^{..} \right\}+\alpha_{i}m_{i}\left\{\frac{u_{yi,t}-u_{yi,t-\partial t}}{\partial t}\right\}=\sum_{j=1}^{n} \left[k_{1ij}\left\{\left(y_{Oi}-y_{Oj}\right)+\left(u_{yi}-u_{yj}\right)\right\}-k_{2yij}\left(r_{i}+r_{j}\right)\right]$ (1.10b)

$m_{i}\left\{\mathop{\frac{u_{zi,t}-2u_{zi,t-\partial t}+u_{zi,t-2\partial t}}{\partial t^{2}}}\limits^{..} \right\}+\alpha_{i}m_{i}\left\{\frac{u_{zi,t}-u_{zi,t-\partial t}}{\partial t}\right\}=\sum_{j=1}^{n} \left[k_{1ij}\left\{\left(z_{Oi}-z_{Oj}\right)+\left(u_{zi}-u_{zj}\right)\right\}-k_{2zij}\left(r_{i}+r_{j}\right)\right]$ (1.10c)

Equations 1.10 (a, b and c) can then be rearranged into equations 1.11 (a, b and c):

(1.10a)

(1.10b)

(1.10c)

The above can be solved explicitly to give u_x,t_ , u_y,t_ and u_z,t_ for every cell at every time step. The updated position will thus depend upon the displacements at the previous two time steps.

This algorithm is run until empirically determined convergence criteria are fulfilled, or for a maximum of 200 iterations. The upper limit is required to allow for situations where the model approaches confluence and the convergence criteria will never be fulfilled. In this case, any residual ‘force’ or overlap is passed back into the agent model, along with the updated cell locations.
