# Supplementary material for: Development of a Three Dimensional Multiscale Computational Model of the Human Epidermis
Source: PLoS One. 2010 Jan 14;5(1):e8511. doi: 10.1371/journal.pone.0008511 (PMC2799518; doi:10.1371/journal.pone.0008511)
Supplement: Appendix S2 — Model Pseudocodes (0.05 MB DOC) [file pone.0008511.s002.doc]

**Appendix S3: Model Pseudocodes**

1. **Cell_agents: Output_Data_to_Message_Board**

**For each** Cell Agent

-Output to the Message board the following memory variables: Agent id, Type, x, y, z, direction, motility, TGF-β1 concentration on the Plasma Membrane

**End**

1. **Check Tiles**

**For each** Tile Agent ‘i’

**If** Tile Agent ‘i’ is a provisional or secondary matrix tile[[1]](#footnote-2)

-ECM Reconstruction_Rules (step 3)

**End**

**End**

1. **Tile_agents: ECM Reconstruction_Rules**

For a certain SM or PM Tile agent ‘i'

**If** one or more Cell agents of type 0 (stem) or 1 (TA) are in physical contact with the tile agent ‘i' **And** the concentration of TGF-β1 on the plasma membrane and in the endosome of at least one such cell agent is larger than 20 mmol/l

**-**New ECM_value for Tile Agent ‘i' = original ECM_value – 0.1[[2]](#footnote-3)

**End**

1. **Tile_agents: Output_Data_to_Message_Board**

**For each** Tile Agent

-Output to the Message board the following memory variables: Agent id, ECM_value, x, y, z

**End**

1. **Cell_agents: Update_Bonds_Rules**

**For each** Cell agent ‘i'

-Number of xy bonds = 0

-Number of z bonds = 0

**For each** Cell agent ‘j’ (i ≠ j)

**If** Euclidean distance between agents ‘i' and ‘j’ is less than or equal to 5

**If** |z_i_ - z_j_| ≤ cell agent radius

-Number of z bonds = Number of z bonds + 1

**Else**

-Number of xy bonds = Number of xy bonds + 1

**End**

**End**

**End**

**End**

1. **Cell_agents: Cycle_Rules**

**For each** Cell agent ‘i'

-Increment Cell agent cycle ticks (i.e. cell progresses in its cycle)

**If** (1) the projected coordinates of cell agent ‘i' on the x-y plane intersects a PM or SM tile **OR** (2) the concentration of TGF-β1 on the plasma membrane of cell agent ‘i’ is larger than 20 mmol/l] **AND** (3) cell agent ‘i’ is a stem or TA cell [(1 or 2) and 3]

-Set cell agent ‘i’ as a cell agent in the active TGF-β1 region

-Set agent ‘i' TGF-β1 Promoter activity level (PRO = 10$\times$(ECM_value + stratification distance) + 20)

**If** Cell agent ‘i’ is (1) a Stem or TA cell **AND** (2) in the mitosis phase of its cycle

**If** Cell agent ‘i’ is (1) a contact inhibited stem cell **AND** (2) not in active TGF-β1 region,

-Set division probability DP to 1%

**Else if** Cell agent ‘i’ is (1) a contact inhibited TA cell **AND** (2) not in active TGF-β1 region,

-Set division probability DP to 2%

**Else if** Cell agent ‘i’ is (1) a Stem or TA cell **AND** (2) not contact inhibited **AND** (3) not in active TGF-β1 region,

-Set division probability DP to 40%

**Else if** Cell agent ‘i’ is a (1) Stem or TA cell **AND** (2) not contact inhibited **AND** (3) in active TGF-β1 region **AND** (4) residing on the substrate on top of PM tile

-Set division probability DP to 2%

**Else if** Cell agent ‘i’ is a (1) Stem or TA cell **AND** (2) not contact inhibited **AND** (3) in active TGF-β1 region **AND** (4) residing on the substrate on top of SM tile

-Set division probability DP to 90%

**Else if** Cell agent ‘i’ is a (1) Stem or TA cell **AND** (2) not contact inhibited **AND** (3) in active TGF-β1 region **AND** (4) stratified (not on the substrate)

-Set division probability DP to 5%

-Generate a uniformly distributed random number R in the interval (0,1)

**If** R ≤ DP

-Add a new cell agent to the complete list of cell agents (the new cell agent will possess some of agent ‘i’ memory variables e.g. type and cell cycle length and some new values e.g. x, y, z and position in its own cycle)

**If** cell agent ‘i’ is a corneocyte **AND** certain distance away from the base membrane

-Eliminate cell agent ‘i’ from the list of cell agents

**END**

1. **Cell_agents: Execute_COPASI_Rules**

**For each** cell agent ‘i’

**If** cell agent ‘i’ is a stem cell or TA cell **AND** (1) in active TGF-β1 region

- Update the executable copasi model with agent ‘i' memory variables (PRO, mRNA, TGF-β1, R1, R2, LRCP, LRCE, R1E and R2E concentrations)

- Externally execute COPASI using the updated model (task: time course simulation)

- Update agent ‘i’ memory variables based on the final results produced by COPASI and stored in a results file

**End**

1. **Cell agents: Can Differentiate?**

**For each** Cell Agent ‘i’

**If** Cell Agent ‘i’ is a Stratified Stem Cell

-Differentiation_Rules (step 9)

**Else if** Cell Agent ‘i’ is a TA Cell certain distance away from the closest stem cell (the distance threshold is higher when a cell is in in active TGF-β1 region)

-Differentiation_Rules (step 9)

**If** Cell Agent ‘i’ is a Commited Cell **AND** (1) certain distance away from the base membrane **OR** (2) surrounded by a certain number of corneocytes (dead signals)

-Differentiation_Rules (step 9)

**End**

**End**

1. **Cell_agents: Differentiation_Rules**

**If** Cell agent ‘i’ is a stem cell

**-**Differentiate into a TA cell

**Else if** Cell agent ‘i’ is a TA cell

**-**Differentiate into a committed cell

**Else if** Cell agent ‘i’ is a committed cell

**-**Differentiate into a corneocyte

**End**

1. **Cell agents: Can Migrate?**

**For each** Cell Agent ‘i’

**If** Cell Agent ‘i’ is a TA Cell

-Migration_Rules (step 11)

**Else if** Cell Agent ‘i’ is a Committed Cell or Corneocyte floating in mid air

-Migration_Rules (step 11)

**End**

**End**

1. **Cell_agents: Migration_Rules**

**If** cell agent ‘i’ is floating in mid air

-Apply a gravitational force towards ECM and iteratively decrease its z coordinate by a small step ∆ until a contact with a another cell or ECM is reached

**If** cell agent ‘i’ is a TA cell **AND** (1) not contact inhibited **AND** (2) has a TGF-β1 concentration on the plasma membrane larger than 10 mmol/l **AND** (3) its closest ECM tile is a PM tile

-Set agent’s ‘i’ migration direction towards centre of such tile using polar coordinates

-Set agent’s ‘i’ migration motility to: 2 $\times$ normal motility (0.1 to 0.5 µm/h)

**Else if** cell agent ‘i’ is a TA cell **AND** (1) not contact inhibited **AND** (2) has a TGF-β1 concentration on the plasma membrane larger than 10 mmol/l **AND** (3) its closest ECM tile is a SM tile

-Set agent’s ‘i’ migration direction towards centre of such tile using polar coordinates

-Set agent’s ‘i’ migration motility to: 1.5 $\times$ normal motility (0.1 to 0.5 µm/h)

**Else if** cell agent ‘i’ is a TA cell **AND** (1) not contact inhibited **AND** (2) its closest ECM tile is a BM tile

-Set a random migration direction for agent’s ‘i’ within ± 45^o^ of its current direction

-Set agent’s ‘i’ migration motility to normal motility (0.1 to 0.5 µm/h)

-New cell x = old cell x + motility$\times$cos(migration direction)

-New cell y = old cell x + motility$\times$sin(migration direction)

**If** cell agent ‘i’ with new x-y coordinates maintains contact with another cell

-Accept new cell coordinates

**Else**

-Reject new cell coordinates and do not migrate

**If** cell agent ‘i’ is floating in mid air

-Apply a gravitational force towards ECM and iteratively decrease its z coordinate by a small step ∆ until a contact with a another cell or ECM is reached

1. **Cell_agents:Output_Data_to_Physical_Solver**

**For each** Cell Agent

Output to the Message board the following memory variables: Agent id, Type, x, y, z, xy_radius, z_radius, integrin and protease concentration (= 0.5 $\times$ TGF-β1 concentration in the cell endosome), direction, motility, TGF-β1 concentration on the Plasma Membrane

**End**

1. A tile agent is a provisional matrix if its ECM_value is between 0.5 and 1 and is a secondary matrix if its ECM_value is between 0.1 and 0.49 (see figure 7) [↑](#footnote-ref-2)
2. When conditions are met, 10 model iterations (i.e. the equivalent of 5 hours in reality) are needed to reconstruct a PM tile with ECM_value = 1 into a BM tile with ECM_value = 0. [↑](#footnote-ref-3)
